# Assessment of Incorrect Surgical Procedures Within and Outside the Operating Room: A Follow-up Study From US Veterans Health Administration Medical Centers

**DOI:** 10.1001/jamanetworkopen.2018.5147

**Published:** 2018-11-21

**Authors:** Julia Neily, Christina Soncrant, Peter D. Mills, Douglas E. Paull, Lisa Mazzia, Yinong Young-Xu, William Nylander, Marilyn M. Lynn, William Gunnar

**Affiliations:** 1 National Center for Patient Safety, Veterans Health Administration, White River Junction, Vermont; 2 Geisel School of Medicine at Dartmouth, Hanover, New Hampshire; 3 Georgetown University School of Medicine, Washington, DC; 4 National Surgery Office, Veterans Health Administration, Washington, DC; 5 Loyola University Stritch School of Surgery, Chicago, Illinois

## Abstract

**Importance:**

Reducing wrong-site surgery is fundamental to safe, high-quality care. This is a follow-up study examining 8 years of reported surgical adverse events and root causes in the nation’s largest integrated health care system.

**Objectives:**

To provide a follow-up description of incorrect surgical procedures reported from 2010 to 2017 from US Veterans Health Administration (VHA) medical centers, compared with the previous studies of 2001 to 2006 and 2006 to 2009, and to recommend actions for future prevention of such events.

**Design, Setting, and Participants:**

This quality improvement study describes patient safety adverse events and close calls reported from 86 VHA medical centers from the approximately 130 VHA facilities with a surgical program. The surgical procedures and programs vary in size and complexity from small rural centers to large, complex urban facilities. Procedures occurring between January 1, 2010, and December 31, 2017, were included. Data analysis took place in 2018.

**Main Outcomes and Measures:**

The categories of incorrect procedure types were wrong patient, side, site (including wrong-level spine), procedure, or implant. Events included those in or out of the operating room, adverse events or close calls, surgical specialty, and harm. These results were compared with the previous studies of VHA-reported wrong-site surgery (2001-2006 and 2006-2009).

**Results:**

Our review produced 483 reports (277 adverse events and 206 close calls). The rate of in–operating room (in-OR) reported adverse events with harm has continued to trend downward from 1.74 to 0.47 reported adverse events with harm per 100 000 procedures between 2000 and 2017 based on 6 591 986 in-OR procedures. When in-OR events were examined by discipline as a rate, dentistry had 1.54, neurosurgery had 1.53, and ophthalmology had 1.06 reported in-OR adverse events per 10 000 cases. The overall VHA in-OR rate for adverse events during 2010 to 2017 was 0.53 per 10 000 procedures based on 3 234 514 in-OR procedures. The most common root cause for adverse events was related to issues in performing a comprehensive time-out (28.4%). In these cases, the time-out either was conducted incorrectly or was incomplete in some way.

**Conclusions and Relevance:**

Over the period studied, the VHA identified a decrease in the rate of reported adverse events in the OR associated with harm and continued reporting of adverse event close calls. Organizational efforts continue to examine root cause analysis reports, promulgate lessons learned, and enhance policy to promote a culture and behavior that minimizes events and is transparent in reporting occurrences.

## Key Points

**Question:**

Are reported rates of incorrect surgery changing in the US Veterans Health Administration (VHA)?

**Findings:**

This quality improvement study found that VHA-reported surgical adverse events have continued to trend downward from 1.74 to 0.47 per 100 000 procedures between 2000 and 2017. In this context, dentistry, neurosurgery, and ophthalmology remain a challenge.

**Meaning:**

The VHA is holding the gains with preventing incorrect surgical procedures while continuing to work in selected areas of continued challenge.

## Teaser

This quality improvement study examines the rate of surgical adverse events in US Veterans Health Administration medical centers from 2010 to 2017 and compares it with rates from previous studies.

## Introduction

Health care continues to strive to improve patient safety. One of the most challenging issues is preventing wrong-site surgery,^1,2,3,4,5,6,7,8^ with 95 wrong-patient, wrong-site, or wrong-procedure sentinel events reported to The Joint Commission in 2017.^9^ This does not include events that may not have been reported to The Joint Commission.^9^ The National Quality Forum considers wrong-site, wrong-patient, and wrong-procedure incidents to be serious reportable events. Accordingly, the National Quality Forum expanded its definitions to be more inclusive, recognizing the need to continue to address this challenge.^10^

Such events have been estimated to occur at a median (range) rate of 0.09 (0-3.9) events per 10 000 cases.^4^ However, these estimates vary, and such rates are difficult to measure precisely.^4,5^ Some rates are based on clinician incident reporting, while others came from closed claims data,^11^ both of which may be limited in accuracy. Furthermore, even with indications of the prevalence of wrong-site surgery, we still need more evidence on why these errors continue to occur and how to prevent them.^4^

The Veterans Health Administration (VHA) National Center for Patient Safety (NCPS) has a national centralized database for reporting patient safety events. The VHA National Surgery Office (NSO) also has a centralized reporting system specifically for surgical patient safety errors.^12,13^ Both systems reconcile these data quarterly to increase the accuracy of wrong-site surgery measurement. The last published^2^ (2006-2009) VHA rate of in–operating room (in-OR) adverse events was 0.4 adverse events per 10 000 procedures. That study also included specialty specific rates and root causes of such errors. The current study contains data on incorrect surgery from the VHA up to 2017. This study is based on data from the largest integrated health care system in the United States with 2 complementary systems reporting for increased accuracy of capturing surgical adverse events. Therefore, this study provides one of the most comprehensive reports on wrong-site surgery, spanning a rate review of 17 years. It not only includes event rates but also provides in-depth information on why such events may still occur and recommendations on how to prevent similar future events.

## Methods

This is a quality improvement study based on the Standards for Quality Improvement Reporting Excellence (SQUIRE) reporting guideline^14^; it describes several years of an initiative to improve surgical safety care. We provide the context and the overall interventions of systemwide efforts to prevent incorrect surgery, results, limitations, and discussion. The Research and Development Committee of White River Junction VHA Medical Center approved this study and the Committee for the Protection of Human Subjects of Dartmouth College considered it exempt. All data were deidentified.

### Time Period and Inclusion Criteria

We analyzed VHA surgical adverse events and close calls occurring between January 1, 2010, and December 31, 2017, reported to NCPS. Incorrect surgical and invasive procedures that occurred within the OR and outside the OR, including procedure rooms, radiology suites, and the bedside, were included. Reports of surgical adverse events could have come from any of the approximately 130 VHA facilities with a surgical program.

### Root Cause Analysis

Reported surgical adverse events in the VHA are reviewed at the local facility. Patient safety managers rate the severity and probability of harm related to events using a safety assessment code (SAC) matrix, developed by the VHA to prioritize adverse events for the root cause analysis (RCA) process.^12^ The SAC score is a combination of severity of harm (catastrophic to minor) and probability of occurrence (frequent to remote), with ratings from 1 to 3 for both actual events and close calls. Events that receive an SAC score of 3 are a combination of the most severe and frequent events.^12^ The most severe cases undergo an RCA, and less severe events are entered as safety reports. Root cause analysis teams examine what happened, why it happened, and how to prevent it from happening again. The data reviewed were limited to information derived from RCAs and safety reports submitted to NCPS and were not abstracted from medical records.

### Critical Incident Tracking Notification

In addition to the RCA and safety reporting, in 2010 the VHA established the Critical Incident Tracking Notification (CITN) process to ensure timely notification of surgical adverse events reported to the NSO and NCPS. The NSO and NCPS reconcile CITN events with RCA and safety report data on a quarterly basis and educate surgical staff in the field by sharing redacted summary reports of these events with the intent of preventing similar events. The CITN-based reporting and lessons learned processes have been described in detail elsewhere.^13^

### Coding

Two of us (J.N. and C.S.) coded 20 reports and reached a coefficient κ of 0.83. Thereafter, the cases were coded independently, and consensus was reached on any items for which the coders had questions. Reports were coded into major categories such as type of event and body segment. They also coded the root causes reported by the teams into groupings such as education and communication. The specific definitions for the coding have been previously described.^1^ For example, close calls were defined as events in which the patient did not undergo a procedure (or have an incision or puncture of the skin, including regional or general anesthesia) that was incorrect. Specific examples of close calls include the wrong patient given a consent form, the wrong procedure written on a consent form, the wrong site marked, or the wrong side of the patient prepared for surgery. As long as the error was discovered and no skin puncture or injection was made, it was a close call.

### Statistical Analysis

A descriptive analysis was conducted in which we summarized the types of adverse events and close calls that occurred, their location, and in which specialties they occurred for the 8-year period between 2010 and 2017. We calculated the rate of in-OR reported adverse events per 10 000 procedures (this was a population-based rate, not sampling, and therefore did not include 95% confidence intervals). The numerator was the number of reported in-OR adverse events over the 8 years and the denominator was the number of surgical procedures performed cumulatively and by surgical specialty in the OR over the study period. We compared adverse event rates with those reported in a previous study (2006-2009).^2^

We performed a trend analysis by modeling the count data of the number of surgical events with a SAC score of 3 using the Poisson distribution (the link function was the logarithm and surgical volume—the denominators for the rates—was the offset) to examine the decrease in the rate of actual events with a score of 3 over time in the VHA. The independent variable was calendar year (modeled as a continuous variable). Rate ratios (RRs) and accompanying 95% confidence intervals were calculated to represent the strength of association between time (year) and incorrect surgery rates, estimated using the Poisson regression. In general, a simple Poisson model has the following general form: log (rate) = intercept + slope × independent variable.

In our trend analysis, we calculated the rate of reported adverse events with the most harm (those with an SAC score of 3) as the numerator over the denominator of the total number of surgical procedures in the OR per year and multiplied by 100 000 for a whole number.

All analyses were performed using SAS statistical software version 9.2 (SAS Institute Inc). All reported *P* values are 2-sided, at a significance level of .05.

## Results

Our review of the most recent period (2010-2017) produced 483 reported cases over 8 years from 86 different VHA medical centers (Table 1). This time frame included 3 234 514 in-OR procedures. Of those cases, 277 were reported adverse events and 206 were close calls. For the adverse events, 172 were in the OR, and 105 were outside the OR (Table 1).

**Table 1.** Ensuring Correct Surgery Reports

Type of Event	No. (%)		
	2001-2006; 5.5 y	2006-2009; 3.5 y	2010-2017; 8 y
Total reports, No.	342	237	483
Reported adverse events	212 (62)	101 (43)	277 (57)
In–operating room adverse events only	108 (51)	50 (50)	172 (62)
Non–operating room adverse events only	104 (49)	51 (50)	105 (38)
Reported close calls	130 (38)	136 (57)	206 (43)

Table 2 displays all 277 reported adverse events (in-OR and non-OR), by specialty and type of event. Ophthalmology had the highest number of reported adverse events (72) followed by dentistry (30) and anesthesiology (28). However, when examining in-OR reported adverse event rates by specialty, dentistry had 1.54, neurosurgery had 1.53, and ophthalmology had 1.06 reported in-OR adverse events per 10 000 cases. Ten of the 13 neurosurgery adverse events were spine cases. Fifty-two of the 86 cases of wrong-implant adverse events were due to completed implantation of an expired lens. No other specialty had more than 1 in-OR reported adverse event per 10 000 cases. This study reported 86 wrong-implant events over 8 years, whereas the prior study (2006-2009) identified 16 wrong-implant events over 5.5 years.^2^ The overall VHA in-OR reported adverse event rate was 0.53 per 10 000 procedures based on 3 234 514 in-OR procedures. This rate is slightly higher than the overall rate of reported in-OR adverse events in the most recent previous study (2006-2009), which was 0.4 per 10 000.^2^

**Table 2.** Reported Adverse Events by Specialty and Type of Event Inside and Outside the Operating Room

Specialty	Adverse Events, No.							
	Wrong Patient	Wrong Side	Wrong Site	Wrong Procedure	Wrong Implant	Wrong Level	Other^a^	Total
Ophthalmology	1	10	2	1	57	0	1	72
Dentistry	0	1	25	1	3	0	0	30
Anesthesiology	0	27	0	0	0	0	1	28
Orthopedics	0	5	4	3	6	1	0	19
Radiology	0	12	1	3	1	1	0	18
Urology	0	9	1	2	2	0	1	15
Neurosurgery	0	2	1	0	0	10	0	13
General surgery	0	2	5	1	3	0	0	11
Dermatology	0	1	11	0	0	0	0	12
Pulmonology or thoracic	0	8	1	1	0	0	0	10
Ear, nose, and throat	1	2	2	0	0	0	1	6
Cardiology	0	1	0	0	4	0	0	5
Plastic surgery	0	1	3	0	1	0	0	5
Podiatry	0	1	1	0	0	0	0	2
Gastroenterology	0	0	0	1	0	0	0	1
Vascular	0	0	0	0	1	0	0	1
Unable to determine	0	8	8	1	8	4	0	29
Total	2	90	65	14	86	16	4	277

^a^ Examples include incorrect medication or anesthetic given, problematic instrumentation used during procedure, and other events not clearly fitting into major categories.

**Figure 1.**
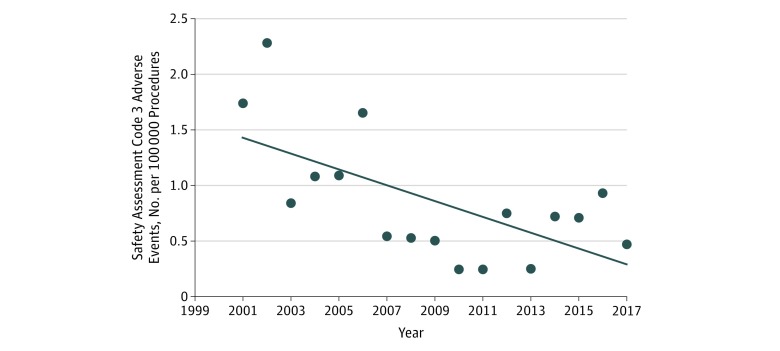
Reported In–Operating Room Safety Assessment Code 3 Incorrect Surgical Adverse Events in Veterans Health Administration Medical Centers, 2001-2017 Points represent the safety assessment code 3 adverse event rate per 100 000 procedures. The line represents the linear trend analysis of the adverse event rate data (*P* = .004).

The rate of in-OR adverse events with the most harm (SAC score of 3) decreased from 1.74 to 0.47 reported adverse events with harm per 100 000 procedures between 2000 and 2017 (includes the 2 previous studies, 2001-2006 and 2006-2009) (Figure 1) based on 6 591 986 in-OR procedures. At the same time, overall reported close calls displayed no clear linear trend.

**Figure 2.**
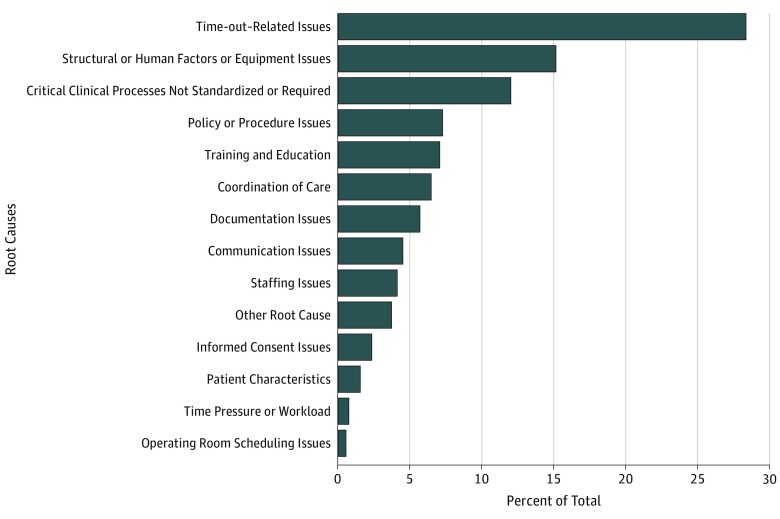
Root Causes for 507 Adverse Events Inside and Outside the Operating Room Events labeled *other* do not fit into other major categories. These include several different types of isolated events, issues with the culture of safety in the facility, or several disruptions or changes to the flow of a procedure.

Table 3 displays type of event by the location of the surgery (in-OR vs non-OR). Figure 2 displays the root causes as determined by RCA associated with the 277 actual adverse events. There were a total of 507 root causes, as events could have multiple root causes. The most common root cause for adverse events was categorized under time-out–related issues (28.4%). In these cases, the time-out was either conducted incorrectly or was incomplete in some way.

**Table 3.** Reported Adverse Event Types Inside and Outside the Operating Room

Event Type	Adverse Events, No.		
	In Operating Room	Not in Operating Room	Total
Wrong patient	0	2	2
Wrong side	41	49	90
Wrong site	25	40	65
Wrong procedure	8	6	14
Wrong implant	79	7	86
Wrong level	15	1	16
Other^a^	4	0	4
Total	172	105	277

^a^ Examples include incorrect medication or anesthetic given, problematic instrumentation used during procedure, and other events not clearly fitting into major categories.

## Discussion

This study focused first on the current update period (2010-2017) and then compared overall rates with serious harm over time during all 3 study periods: 2001 to 2006,^1^ 2006 to 2009, and 2010 to 2017.^2^ We show a downward trend (2001-2017) in reported adverse events with the most harm, with continued reporting of close calls. This supports that improvements are likely not due to decreased reporting overall. Continued close call reporting also indicates a culture of safety, supporting learning from events.

Beginning in 2010, the NSO collaborated with the NCPS in establishing the CITN process for immediate reporting of surgical adverse events. The number of reported surgical adverse events increased in comparison with previously reported VHA studies on this topic.^1,2^ For example, this study reported 86 wrong-implant events over 8 years, whereas the prior study (2006-2009) identified 16 wrong-implant events over 5.5 years.^2^ An event such as implanting an expired lens may previously have gone unreported, but the CITN process clearly mandated it as a reportable event. Accordingly, the overall rate of reported in-OR adverse events increased from the level shown in the 2006 to 2009 study^2^ (0.4 to 0.5 per 10 000 procedures) in the most current period. However, even this rate remains low and well within the private-sector range of 0 to 3.9 reported surgical adverse events per 10 000 procedures.^4^

When reported in-OR adverse events were analyzed as a rate, we found that dentistry and neurosurgery had the highest rates of reported adverse events, followed by ophthalmology. Ten of the 13 neurosurgery adverse events were spine cases. The July 2013 update of the VHA Ensuring Correct Surgery and Invasive Procedures^15^ directive added greater specificity regarding the intraoperative requirements for the attending physician performing spine procedures to confirm the placement of spine marker with a fixed radiograph to locate the appropriate vertebral body or intervertebral space for the procedure. The policy is currently being modified to recommend that the location of the spinal marker must be confirmed with a radiologist if any member of the surgical team questions the location of the spinal marker. Devine et al^5^ wrote extensively about the challenges that clinicians have with wrong-level spinal surgery.

We were able to calculate a specific rate for in-OR reported dental events in this review because we had denominator data specifically for oral surgery, which we did not have in the last review (2006-2009).^2^ In the last review, dental events were one of the top 5 specialties to report adverse events, and dentistry was in the top 4 this time; this rate may not signal a change in the frequency of dental events. However, we are working closely with VHA dental leaders to clarify that sites must be marked using digital imaging, radiographs, or intraoral charts.

Ophthalmology had the second highest rate of reported in-OR adverse events. This is similar to our previous reports (2001-2006 and 2006-2009).^1,2^ Fifty-two of the 86 cases of wrong-implant adverse events were due to completed implantation of an expired lens. The Joint Commission does not include expired implants as a type of wrong-site surgery.^6^ However, because of the risk of causing harm, the VHA includes expired implants in its definition, and these 52 events are a warning sign indicating failure to follow policy. Specifically, the VHA modified directive will include a requirement to specifically state the implant expiration date with a read-back for lens implants during the time-out and just prior to implantation.

Reducing eye surgery adverse events has been a challenge. The cases are short, the time pressure is high, and there are multiple steps in the process from measuring the needed lens strength in the clinic to implanting a nonexpired correct lens in the OR. We issued a patient safety alert on this topic, conducted an in-depth assessment of the challenges,^16,17^ and discussed further possible actions during a VHA summit to prevent incorrect surgical adverse events in December 2017. Others have also shared similar challenges with incorrect eye surgery.^18^

Overall, the most common root cause for incorrect surgery was time-out–related issues. This differs from the last VHA report, in which a lack of standardization of clinical processes was the top root cause. This may signal progress, as sometimes in RCAs the team will write that although a time-out was done, it was not done mindfully. This signals a stronger commitment to safety rather than just ambivalent compliance.^19^

There are many possible reasons for the improvements in this report and for the challenges that still exist. There has been a steady focus on OR safety in the VHA. We have continued to produce and share the surgical lessons learned and to collaborate closely with the NSO.^13^ Each quarter we jointly review all reported adverse events for surgery, develop lessons learned, share these on a conference call with surgical leadership from the VHA’s 18 regions (Veteran Integrated Service Networks), and distribute summary information to staff.^13^

### Limitations

This study has limitations. First, these data are based on self-report and therefore we cannot be sure all incorrect surgical adverse events were included, particularly those that occurred outside the defined structure of the OR. We also do not have demographic data about patients because the RCAs and safety reports are deidentified. In addition, there were times the reports did not contain enough information to categorize the cases fully. The coding of harm is done at the local facility and there may be variation in the application of the SAC scoring system.

## Conclusions

We have demonstrated an overall decrease in the number and severity of incorrect surgical procedures in the VHA. Telling people to follow the policy is not enough to prevent incorrect surgery. We need continued leadership support and peer-to-peer communication such as sharing lessons learned. We need to ensure the use of standardized procedures and to initiate specialty-specific safety measures in areas such as for eye and spine surgery. Most importantly, we need to do more than go through the motions of a time-out. We need to mindfully conduct time-outs, use the universal protocol, and use human factors engineering to work toward eliminating incorrect surgical adverse events.
